# Response to: Neuromelanin? *T*_1_ shortening is a necessary condition

**DOI:** 10.1093/braincomms/fcag215

**Published:** 2026-06-09

**Authors:** Jean-Baptiste Pérot, Marta Gonzalez-Sepulveda, Miquel Vila, Stéphane Lehéricy

**Affiliations:** Paris Brain Institute—ICM, MOVIT team, Sorbonne Université, Inserm U1127, CNRS 7225, Hôpital Pitié-Salpêtriere, Paris 75013, France; Department of Radiology, Lausanne University Hospital (CHUV), Lausanne 1011, Switzerland; Neurodegenerative Diseases Research Group, Vall D’Hebron Research Institute (VHIR)-Network Center for Biomedical Research in Neurodegenerative Diseases (CIBERNED), Barcelona 08035, Spain; Neurodegenerative Diseases Research Group, Vall D’Hebron Research Institute (VHIR)-Network Center for Biomedical Research in Neurodegenerative Diseases (CIBERNED), Barcelona 08035, Spain; Department Malalties Neurodegeneratives, Catalan Institution for Research and Advanced Studies (ICREA), Barcelona 08036, Spain; Paris Brain Institute—ICM, MOVIT team, Sorbonne Université, Inserm U1127, CNRS 7225, Hôpital Pitié-Salpêtriere, Paris 75013, France

We thank Dr Watanabe for some useful comments^1^ regarding our article by Pérot *et al*.^2^ and the origin of nigral hyperintensity signal on the sequence commonly called neuromelanin-sensitive MRI or NM-MRI.^3^ NM-MRI has demonstrated usefulness for the study of neurodegeneration of the substantia nigra (SN). NM-MRI provides markers (reduced volume and signal hyperintensity) capable of detecting SN neurodegeneration in Parkinson’s disease with good accuracy, tracking the progression of lesions with disease worsening, and which are correlated with the severity of clinical signs and striatal dopaminergic function.^4^ Using this sequence, the area of high signal intensity has been associated with the presence of neuromelanin containing dopaminergic neurons in the substantia nigra pars compacta (SNc) in histological studies.^4^ However, the exact origin of the signal hyperintensity and its relationship to neuromelanin are still debated, which calls into question the sensitivity and specificity of NM-MRI to neuromelanin.

As mentioned by Dr Watanabe,^1^ the T_1_-weighted turbo spin echo sequence used in^2^ is indeed a T_1_-weighted sequence with a magnetization transfer (MT) effect. Even without application of an explicit MT preparation pulse, studies have shown that this sequence is sensitive to intrinsic MT effects due to off resonant radiofrequency fields from adjacent slices.^5^ This is clearly visible, for example, when comparing single-slice (Fig. 1A) and multi-slice (Fig. 1B) acquisitions of the sequence with the same parameters. Images clearly show the expected T_1_ contrast in the single-slice sequence (without MT effect) and the contrast enhancement of the SNc due to MT effect on the multi-slice sequence where signal in the white matter is more suppressed than in the grey matter. This confirms that, in the NM-MRI sequence, MT effects contribute significantly to contrast mechanisms.

**Figure 1:**
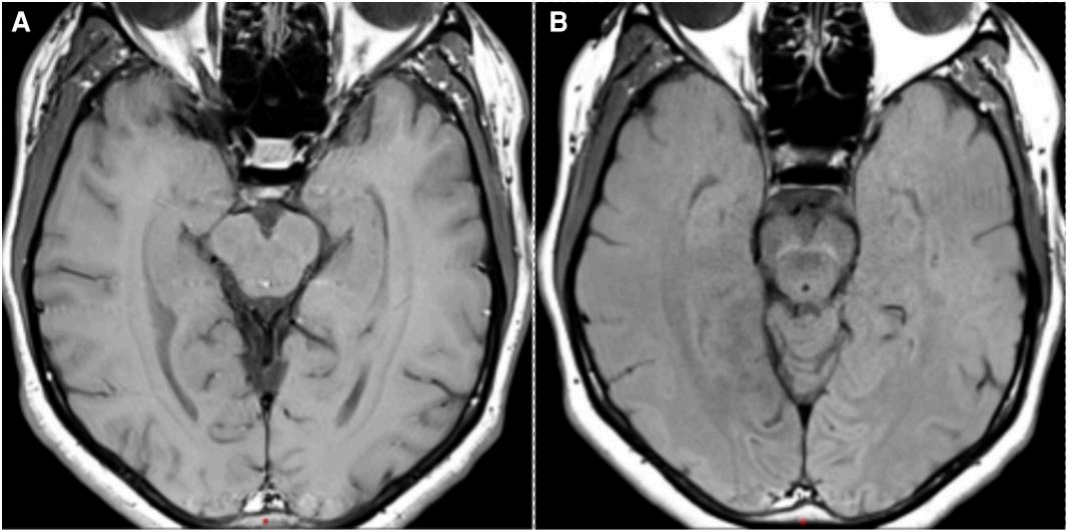
**Illustration of incidental magnetization transfer effect.** (**A**) Single-slice and (**B**) multi-slice Turbo Sin echo T1-weighted sequence without magnetization transfer preparation (TR: 890 ms, TE: 13 ms, three averages).

Nevertheless, our experiment in the animal model at 11.7T shows that the progressive accumulation of neuromelanin after injection of AAV-hTyr, in a species where it is naturally absent, led to a progressive increase in T_1_ signal intensity and a decrease in T_1_ relaxation time. This effect was specific to the injected SN and not observed in other regions. For instance, at 1 month post injection (1mpi), no effect on T_1_ was observed in the striatum (T_1 Str_ = 2.1 ± 0.4 s before injection and 2.0 ± 0.1 s at 1mpi, *P* = 0.23) or the contralateral SN (T_1 SN contra_ = 2.1 ± 0.2 s before injection, 2.1 ± 0.1 s at 1mpi, *P* = 0.09), whereas T_1_ was significantly reduced in the injected SN (T_1 SN ipsi_ = 2.1 ± 0.3 s before injection versus 1.8 ± 0.1 s at 1mpi, *P* < 0.001). In favor of a contribution of neuromelanin, studies have shown that although metal-free neuromelanin has a negligible effect on MT, the neuromelanin–iron complex produces T_1_-shortening effects that could contribute contrast in NM-MRI acquisitions,^6^ in physiological concentrations.^7^ Iron in the SN is stored in ferritin and neuromelanin. Ferritin is the primary storage mechanism in the SN pars reticulata whereas neuromelanin is the primary storage mechanisms of iron in the SNc.^7^ This was also shown in a combined MRI—histology study, reporting colocalization of neuromelanin and R_2_* signals (an indirect measure of iron concentration) in the SNc.^8^ In Pérot *et al*.,^2^ we found a progressive increase in magnetic susceptibility in the ipsilateral hyperintense SN, suggesting iron accumulation, probably in the form of neuromelanin–iron complexes. In humans, studies of the neuromelanin signal during ageing have also shown a progressive increase in T_1_ signal with age in the SN, up to a maximum around 50–60 years, followed by a progressive decrease beyond this age,^9^ a kinetic profile which follows that of histological observations of neuromelanin concentration in the SN.

However, the association between neuromelanin build-up and T_1_ reduction does not necessarily imply that neuromelanin, or rather the neuromelanin–iron complex, is the direct cause of the T_1_ changes. The effect could indeed be due to other abnormalities associated with neurodegeneration, such as neuromelanin accumulation in granules, distribution of free versus bound water and neuronal density. As suggested previously, the increased signal intensity in the SN may not be ascribed to neuromelanin but to higher proton density and lower macromolecular fraction.^10^ In hTyr-overexpressing rats, neuromelanin progressively builds up in dopaminergic neurons until it occupies a major portion of the neuronal cytoplasm. Neuromelanin accumulates in autolysosomes, including pigments, proteins and lipids, transforming a vesicle containing macromolecule-rich fluid (like a lysosome) with high concentrations of water bound to macromolecules into an autolysosome filled with neuromelanin containing a large amount of melanic pigments, lipids (hydrophobic) and misfolded proteins.^11^ This can alter the local water distribution, decreasing macromolecule-bound water and increasing free water resulting in increased signal intensity in the injected SN during the early phase post-injection. Beyond 2 months post-injection, the loss of melanized cells would equalize the macromolecular/free water ratio between the background and the SN and would contribute to the reduction in SN signal intensity.

Dr Watanabe highlights that other brain regions besides the SN are hyperintense with the NM-MRI sequence,^1^ which would support the lack of sensitivity of this sequence to neuromelanin. However, mechanisms of high signal intensity in other grey matter regions in the hTyr rats may not be identical as the one in the SN. For instance, in the hTyr rats at 1mpi, the cell layer of hippocampus had longer T_1_ (2.2 ± 0.2 s) and lower macromolecular proton fraction (MPF = 0.068 ± 0.004) than the injected SN (T_1_ = 1.8 ± 0.1 s, MPF = 0.071 ± 0.009) suggesting that contrast mechanisms between these two regions may differ.

Regarding Fig. 4A, we thank Dr. Watanabe for noticing the error concerning the R_1_ map. The image shown in Fig. 4A was indeed a T_1_ map. This image has been replaced by the correct R_1_ map in the article.^2^ We wish to emphasize that this error was only in the figure and that the R_1_ values used in the manuscript and in Fig. 4D were indeed extracted from the R_1_ maps and that the results are unchanged.

Lastly, we proposed a parallel between hTyr rats and NM-sensitive data obtained in a human cohort of early or prodromal patients with Parkinson’s disease and healthy controls.^2^ Although we cannot rule out that the contrast mechanisms between the animal model and humans differ in certain aspects, we have hypothesized that neuromelanin accumulation and neurodegeneration are likely explanations for the increase and decrease in contrast over time, respectively, in patients and controls. The respective contribution of MT and T_1_ effects will need to be investigated in both patients and controls. For instance, quantitative multiparametric mapping analysis, combining measurements of T_1_, T_2_*, proton density and MT ratio, will allow determine the respective role of NM-iron and macromolecular water fraction in SN signal changes in Parkinson’s disease using NM-MRI.

## Data Availability

Data sharing is not applicable to this article as no new data were created or analysed.
